# Polycentric and resilient perspectives for governing the commons: Strategic and law and economics insights for sustainable development

**DOI:** 10.1007/s13280-022-01719-x

**Published:** 2022-05-31

**Authors:** Andrea Gatto

**Affiliations:** 1 Wenzhou-Kean University, Wenzhou, 325060 Zhejiang China; 2 Natural Resources Institute, Central Avenue, University of Greenwich, Chatham Maritime, ME4 4TB UK; 3 Centre for Studies on Europe, Azerbaijan State University of Economics (UNEC), Baku, Azerbaijan

**Keywords:** Common-pool resources, Cooperative game theory, Elinor ostrom, Regulation, Rodotà commission, Water governance

## Abstract

Commons governance theory is central to identifying and managing conflicts arising from natural and cultural resources traps. Scholars – using game theory and economic analysis of law – have proposed alternative models, consisting of a set of mitigated scenarios, multiple players, and new equilibria in commons governance. Likewise, novel legal innovations of the commons have also been designed. Reinterpreting the commons in light of political economy, ecology, and pluralistic approaches, a critical review of existing scholarship, economic analysis of law, and case study investigations are performed. Examining an array of views – including governance of water in Ecuador, Bolivia, and Italy – a research and policy agenda is put forward to offer original interpretations and novel holistic perspectives. Germane environmental policy implications deriving from SDGs, resilient governance, and polycentric perspectives are thus extrapolated. Finally, pluralistic frameworks drafted by mitigation and adaptation are measured by improved sustainable development performance in commons, resource, and water governance.

## Introduction

Defining the commons is necessary to determine sound policies that attempt to provide effective solutions for governing natural and cultural resources (Ostrom, 1990, 2010a). The governance of common-pool resources (CPR) can yield a large range of provisions that are today paramount in the field of international cooperation, sustainable development, environmental, resource, and energy governance, as well as climate change adaptation (Ostrom et al. 1993; Agrawal 2001; Ostrom 2010b; Paavola 2011; Patt 2017). In the last half-century, two publications benchmarked the scientific debate: Garrett Hardin’s *Tragedy of the commons* (1968), which pointed out the impossibility to acquire optimal management of the common resources; and Elinor Ostrom’s *Governing the commons* (1990)*,* that furnished novel scholarly and practical solutions.

In the most recent works from Elinor Ostrom and further scholars, game theory became crucial to determine such solutions. In terms of methodology, game theory has always played a determinant role in outlining the characteristics of the commons governance, proposing robust solutions (Ostrom 2010a). Little space has been given to date to the role of law and economics. Ostrom aimed to resolve the conflicts arising from the management of common utility goods within confined communities—a typical feature of common-pool resources governance (Ratner et al. 2013). For achieving this scope, a set of rules for self-governance were proposed (Ostrom 2008). These rules were deemed necessary for progress in economic, social, and environmental governance, in addition to sustainability outcomes (Ostrom et al. 1993). In this context, game theory provided strategic opportunities to understand these conflicts, with the goal of optimizing the shared use of natural resources, ensuring long term economic viability (Ostrom 1990).

Regulation has been demonstrated to be paramount in this discourse, especially where practical, legal implications arose. On one hand, it has been shown that self-governance demands a tailored set of stringent rules (Ostrom 1990, 2001). On the other hand, to let polycentric and adaptive governance work, “peaceful contestation and enforcement of a shared system of rules” is required to guarantee “the attribute of continuous competition and conflict resolution” (van Zeben 2019a). These features signal a pivotal role for resilience, in a framework designed for the sustainable governance of commons and natural resources (Ostrom et al. 1993; Agrawal 2001; Gatto 2020).

In the last few years, practical implementation within the legal systems were characterized by the proliferation of a wide set of proposals for legal reforms composed of several constitutions targeting the commons (Wolkmer 2018a, b). The work at hand stresses three case studies: one from the Italian legal system, and two from the Ecuadorian and Bolivian constitutions. In Italy, the reforms did not find a final implementation during the proposals of constitutional reform drafted by the Rodotà Commission (Commissione Rodotà 2007). In Latin America, on the other hand, riding the wave of *constitucionalismo andino*, entire sections of the new constitutions were adopted, wherein both the commons and governance were succinctly defined (Melo and Gatto 2014). In these constitutions, led by the *buen vivir* (i.e. well-living) and *vuelta biocentrica* (namely, biocentric turn) principles, the commons governance and benefit were crucial (Gudyanas 2009).

In this context, it becomes necessary to pursue diversified approaches. Considering multiple angles could enable adaptive, learning, and scattered governance at different levels—i.e. polycentric governance (Ostrom 2010b; van Zeben 2019a). In this way, polycentricity becomes a determinant step to ease an adaptive, mitigated, and reflexive governance agenda (Ostrom 2010a, b) that would favor governance-based resilience. In return, reaching these goals would allow for an optimized resilience of natural resource governance that would result in increased sustainability of the complex system (Anderies et al. 2013; Melo and Gatto 2014; Gatto and Drago 2020a, b).

This perspective paper aims to critically examine the commons governance. The political economy behind the transition from selected strategic approaches to outlooks based on the economic analysis of law is explored. In this study, governance is a principal player of sustainable development along with the economy, society, and the environment. Taking a pluralistic look, this inquiry’s objective is to examine diverse perspectives from existing scholarship and legal doctrine, and to elicit fresh insights. To date, this is the first attempt to combine cooperative game theory and economic analysis of law on the commons with a focus on Latin America, Italy, and water governance. Making use of this blended interpretation, the paper offers an original perspective on the commons governance.

**Fig. 1:**
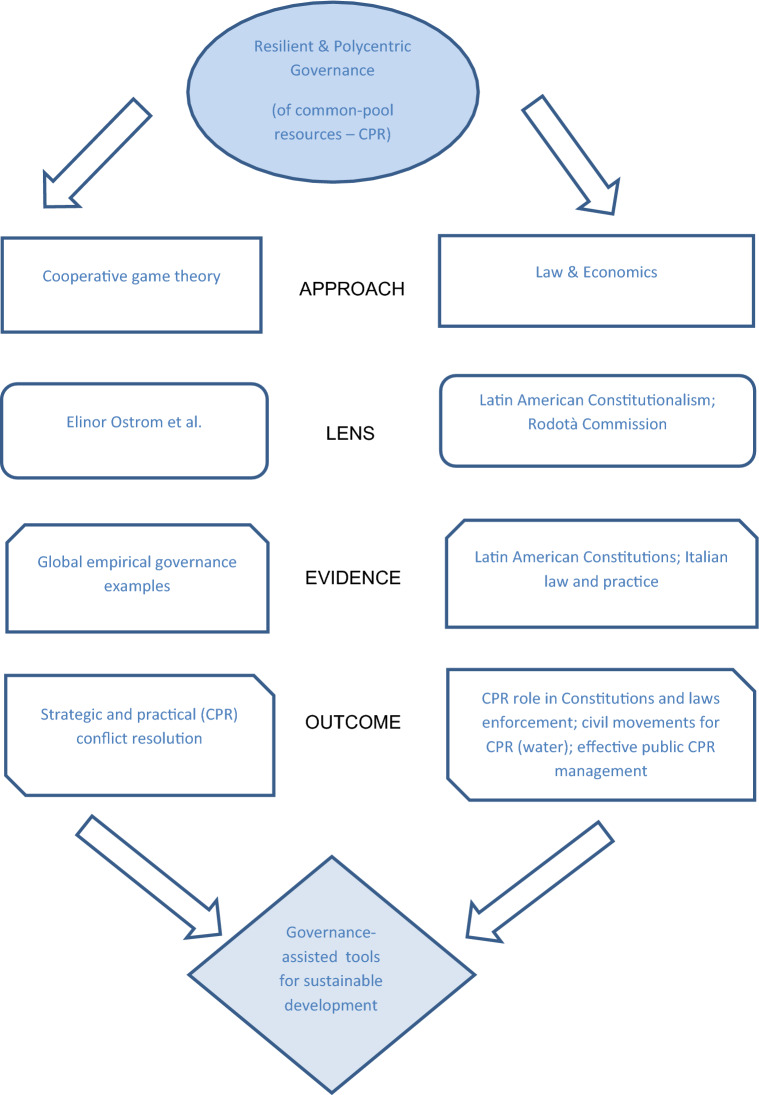
Resilient and polycentric CPR governance for sustainable development: rationale

Figure 1 plots this paper’s angle, based on resilience and polycentrism. Figure 1 depicts the combined twofold outlook and intended governance-driven sustainable development outcomes. This diagram has the intention to clarify the contribution of diverse resilient and polycentric governance perspectives to reach governance-assisted sustainable development. In this view, commons are crucial. The key elements and research’s rationale are as follows:

1. The *approaches*—cooperative game theory; and law and economics.
2. The *lens*—Elinor Ostrom et al.; viz. Latin American Constitutions and Rodotà Commission.
3. The *evidence*—on one hand, global empirical governance examples; on the other hand, Latin American Constitutions and Italian law and practice.
4. The *outcomes*—made up of strategic and practical (CPR) conflict resolution; and CPR role in Constitutions and laws enforcement, civil movements for CPR, and effective public CPR management.

After defining the commons within the categories existing in Public Economics, the paper draws the theoretical framework of the commons. A review is conducted over the different theories implemented on the commons, attributing an emphasis on Elinor Ostrom—and related scholars'—theses. Therefore, the bill of law drafting proposed by Commissione Rodotà in Italy and the Constitutions of Bolivia and Ecuador are explored along with the case studies of water. The Discussion proposes a baseline for the commons governance, where resilience and polycentricity are detected as principal assets to ensure sustainable natural resource uses. Thus this paper presents solutions, policy actions to be undertaken, and prospects for addressing the sustainable development agenda.

## What are commons?

### Defining the commons

*Commons are goods of the natural and cultural heritage of common utility, which are primarily used to address a fundamental right*. Usually, commons are governed by the public or private sector. Commons include: natural resources, areas of fishing, air, along with balanced governance of food, water, energy, and minerals, in addition to environmental preservation (see Hardin 1968; Ostrom 1990). Commons are non-individual, non-excludable, and rival goods. The use of a commons by an additional consumer creates a decrease of the good and an inefficient distribution of the beneficial rights. A tariff on use may lead to information asymmetry in addition to free-riding, potentially giving way to overuse or arbitrarily undue use of the good. Table 1 classifies the goods.

**Table 1:** Classification of commons and other types of goods

Rivalry excludability	High	Low
Yes	Private Goods	Club Goods
No	Commons	Public Goods

### A cooperative strategy

In the last few decades, commons governance theory has frequently exploited game theory to find solutions to reconcile conflicts. It is possible to translate the theory and the empirical evidence defended by Elinor Ostrom in strategic form. In this game "*herders themselves can make a binding contract to commit themselves to a cooperative strategy that they themselves will work out*" (Ostrom 1990). The game is framed in a cooperative matrix. A binding contract is enforced by an external counterparty. This way, the players will be required to negotiate the limit of the field capacity, the execution costs of the agreements, and to find a deal that provides a fair distribution of costs and benefits before making their move.

**Fig. 2:**
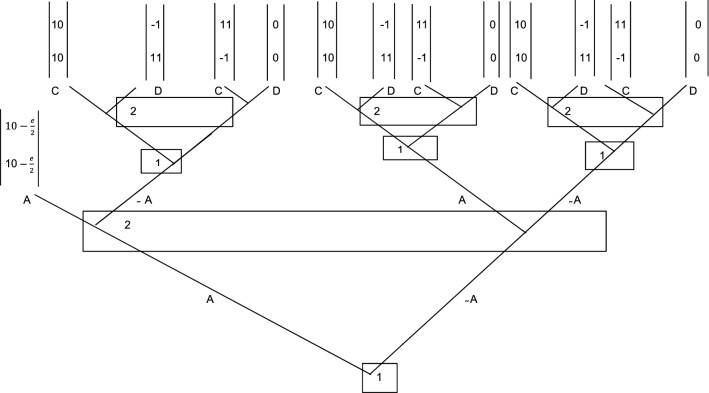
Self-financed contract-enforcement game

As far as information is concerned, unlike games with a central authority, the player will no longer depend on the action of a distant central government and will implement the contract itself. At the same time, the player will reject any contract proposal with incomplete and partial information. They will be likely to have better information about a central government than the ability and the actions of the second player will be able to establish a more effective sanction system. The player will also be more motivated to monitor the counterpart, generating savings on any external monitoring costs. Hence, the contract will be submitted to the authority and signed only upon prior consent. Likewise, any request for execution cost that exceeds the difference between cooperative strategy and non-cooperative strategy, by both the players, will be rejected. It should now result clearly that the dominant strategy contemplated in this game will be the cooperation of both players (10.10)—that is where the strength of Ostrom’s formulation lies. Figure 2 sketches the equilibrium presented.

### Solutions from law and economics

Law and the economic analysis of law can help to protect fundamental rights stemming from the commons enhancing the social, economic, and environmental dimensions. Moreover, the economic analysis of law can be used from different angles to operate quantitative and qualitative analyses. This includes microeconomic, policy, regulation, and constitutional explorations of social-environmental governance management and sustainable development.

Several of the new Latin American constitutions attempted to build a bridge between lawmaking and policymaking, putting into practice the commons theory and sustainable development mandate (Gudynas 2011). Specifically, the Ecuadorian Constitution of 2008 and the Bolivian Constitution of 2009, both integrated the rights to natural commons and the right to cultural commons into their respective legal systems. These constitutions have been represented from pluricultural state, diversity, and interculturality; forging a novel, real-life relationship with sustainability. After all, “Commons are essential to life and to the well-living in harmony with nature” (Constitution of the Republic of Ecuador 2008). This ecological transition, whereby nature takes the lead with respect to the socio-economic sphere, is intended to be driven by the indigenous populations. Herein lies the most innovative attribution to the vulnerable groups destined by this piece of law. On top of that, this outlook represents one of the most notable legal openings to pluralism.

When dealing with commons, the law is not necessarily enforced and governance is often ineffective (Fantini 2020). That is why conjoint political, economic, and legal analyses are of foremost utility—and law and economics applications can assist this need. Within commons governance, water is of particular importance (Zwarteveen et al. 2017). Water is crucial for human life and has been declared a human right by the United Nations (UN General Assembly 2010). However, all over the world, access to water and water security are not always guaranteed. For this reason, it is important to study the economic practices of industrialized economies. Such as water governance in Italy, which led to the water movements and the implementation of an ad hoc technical commission of experts—mostly public law and law and economics specialists—to promote new laws for protecting the commons (see Commissione Rodotà 2007). This bottom-up movement generated the proliferation of a wealth of scholarship on the commons and water tutelage. Eventually, research and law practice inspired the creation of the commons department within the municipality of Naples as well as the municipalization of its water utility (Carrozza and Fantini 2013).

### Commons, governance, and sustainable development

Although commons are not explicitly mentioned in the Agenda 2030, the role of natural resources and their governance is a priority to reach sustainable development goals (UN 2015). Intergenerational equity—through social and environmental justice—is necessary to ensure similar outcomes across various generations despite contemporary changes (Keeble 1988; Bailey et al. 2014; Gatto and Cisco 2021). Natural resource governance is also included in SDGs (UN 2015). Goal 11 recommends reaching inclusive, safe, resilient, and sustainable human settlements; whilst Target 11,7 prescribes universal access to green and public spaces—especially for the destitute and the vulnerable. The proposed indicators are remarkable in policy terms: indicator 68 attempts to measure land consumption rate to population growth rate ratio; while indicator 70 measures the area of public and green space over total city space.

Governance can be considered the forgotten dimension of the sustainability framework designed by Meadows et al. (1972) in “Limits to growth”—besides the economy, the society, and the environment. (Resource) resilience definition is still flawed and non-univocal though it can be interpreted as the capacity to learn and adapt in face of change (Gatto and Drago 2020a). Meanwhile, novel metrics are increasingly providing a sound foundation to define and quantify resilience policy in resource governance domains (Aldieri et al. 2021).

Given these conditions, multilevel governance emerged (Chaffin et al. 2014). Resilience governance represents a method for drafting improved natural resource use (May 2018; Rowland 2017; Oliveira 2015). Resilience is conceived as the governance of complexity; and it is applicable at the local, national, and international levels (Chandler 2014). Resilience needs actions at the economic and legal level, be it ethics, sustainable development, cooperation, or politics—paramount to respond to shocks, adverse and major natural events or continuous change (Georgescu-Roegen 1993; Holling 1996). Resilience governance emerges from its interconnectedness with sustainability and vulnerability adaptation, as well as mitigation and its potential contribution to natural resource governance (Ostrom 2001; Gatto and Busato 2020).

### Adaptive, reflexive, and polycentric governance

The shaped framework recalls bottom-up governance, where communities are the agents of change for the commons governance (Gatto and Sadik-Zada 2021). A notable example is represented by renewable energy communities evidence. This is sustained by policy strategies whose aim is to provide reliable energy access and boost entrepreneurship—ensuring women and the otherwise vulnerable have routes to empowerment (Gatto and Drago 2021). Another approach for governing change is represented by reflexive governance (Feindt and Weiland 2018). In this conceptualization, societies and institutions can represent the preparedness drivers in response to ecological vulnerability and degradation. The model aims to provide resilient infrastructures by making use of participatory, experimental, and learning processes (Ostrom 2010b).

Social-ecological systems also benefit from adaptive and anticipatory governance. In this scheme, systems can adapt to abrupt change—considered as crises—due to improved learning environments characterized by cooperative adaptive comanagement, social networks implementation, and self-organization as well as self-governance (Ostrom 1990; Ostrom et al. 1993; Folke et al. 2005; Stockholm Resilience Centre 2012; Boyd et al. 2015). This feature offers remarkable nexuses to polycentricity, since polycentricy relies on individuals’ ability to self-govern (van Zeben 2013, 2019a). In the polycentric model attributes and institutional essentials (Ostrom 1972) depend on the existence of three prerequisites—namely access to information, capacity to learn, and access to justice (van Zeben 2019b). This speculation originates from Vincent Ostrom’s thesis, stating the need for polycentrism to access to “information and the ability to learn in achieving a self-governing society” (Ostrom 1999). The commons governance passes through the implementation of institutions bearing adaptive capacity related to societal and environmental change (Gupta et al. 2010). Historically and socially, institutions have been a core part of processes and debates (Pierson 2011). Their role is crucial when it comes to environmental governance (Young 2003) and consequently, the quality of resource management (Sadik-Zada 2016). Institutions have a direct function in promoting sustainability (Agrawal 2001) as well as resilience policies and outcomes (Sjöstedt 2015). Polycentric governance has practical applications also for spatial development, territorial sustainability, and land preservation, with the scope to avoid land degradation (Orchard and Stringer 2016; Lanfredi et al. 2022). In the EU, polycentric spatial planning has become a concrete intervention tool for the policy agenda (Davoudi 2003; De Rosa and Salvati 2016).

## Evidence from Latin America and Italy

### The legal frameworks

In the last few years, Italian political will has grown such that lawmakers proposed a set of rules to enhance the governance of the commons through legal and institutional methods (Rodotà 2013). A recently proposed bill drafted law that defined new categories of goods (Commissione Rodotà 2007). The draft law was eventually promulgated by ministerial decree and was thus assigned in 2007 to the Rodotà Commission in order to formulate novel definitions of sovereign, in order to formulate novel definitions of sovereign, social, and common goods. The Commission—led by the prominent jurist Stefano Rodotà—was the culmination of a broad social movement seeking to reform sections of the Italian Civil Code. The Commission intended to provide a new legal framework for property rights (Bailey and Mattei 2013).

Despite its vast potential impact, the bill proposal did not reach the parliamentary debate. The reform would have rejuvenated obsolete categories pertaining to Italian law,^1^ and the Italian Constitution itself (Commissione Rodotà 2007; Lucarelli 2011; Melo and Gatto 2014). Though the Italian Legislative Reform drafted by the Rodotà Commission still remains a legal benchmark initiative—a blueprint for cooperation, development, and sustainability—that face urgent, compelling policy issues.

The proposal was part of a broader movement that sought to lay the foundation for a more just governance of the commons. The process would have provided novel Public Law solutions. This would have facilitated the "public-to-common" transition and new interpretations of natural and cultural resources (Lucarelli 2011). The public-to-common-theory would have promoted the tutelage of sovereign goods, social goods, and commons; while fostering active citizenship, democratic participation, and the protection of fundamental rights (Commissione Rodotà 2007; Lucarelli 2010, 2011; Melo and Gatto 2014).

To emphasize recent legal evidence in the field of commons governance, it is worth underscoring the emergence of another international legal innovation: the *constitucionalismo andino* (Andean constitutionalism). Born out of four decades of promulgation of new constitutions that marked a new wave of legal innovation in Latin America.

The Bolivian Constitution, for example, interprets the commons as a social and economic right. Art. 33 foresees the right to a healthy and harmonious environment; whereas art. 35 and 46 state the right to health, social security, and work. Moreover the commons plays a crucial role in the Bolivian Constitution: natural environmental commons deserve conservation, protection, and regulation (art. 342), as do forests, subsoil, and biodiversity (art. 348, 380), water resources (art. 373), and the Earth resources (art.393). The same protections and regulations also apply to the commons belonging to the Bolivian Amazon (art. 390–392), in addition to sustainable agricultural development (art. 405–409) (Constitution of the Plurinational State of Bolivia 2009).

Meanwhile, the well-living principles are established in Chapter 7 of Title II—art. 12–34 and 340–394 of the Ecuadorian Constitution. Wherein the *buen vivir* assumes a crucial role, especially when related to sustainable development and sustainability (Gudynas 2009). The Ecuadorian Constitution proposes a direct defense of the commons through articles 395–415, which protects biodiversity, outlines the rights of nature, and other pivotal elements to live in harmony with nature. Moreover, several constitutional articles are devoted to outlining a policy framework for water (art. 12), nutrition and a healthy environment (art. 13–14), safe and healthy habitats and dwellings (art. 30), health (art. 32), cities and public spaces, work, and, finally, security (Constitution of the Republic of Ecuador 2008).

### The transition towards commons governance in practice: The case study of water governance

Water governance can be used as a case study of the path outlined in this inquiry. Water is, indeed, trailblazing the transition towards more sustainable, resilient, and participative governance models. One can refer to the previously explored pieces of evidence—Italy and Latin America—to elicit some applied results from the field. In this sense, the case studies of water governance in Latin America and Italy are instructive (Gusmai 2018).

#### Water governance in Latin America

The new Latin American constitutionalism proposes a novel ecological paradigm. Food security and water management are paramount in the transition towards more sustainable resource governance and the mitigation of anthropogenic impact (Wolkmer and Venâncio 2017). Through a bottom-up, pluralistic outlook, the new constitutions shape authentic biocentric ethics where the right to water plays a primary role (Wolkmer et al. 2012; Wolkmer and Wolkmer 2021).

The right to water is truly pronounced in Latin American constitutions. Indeed, all living creatures are intended as part of the ecosystems in an intergenerational change vision based on sustainable development (Wolkmer et al. 2012). These constitutions also stress commons protection in a bid to outline socio-environmental conflict resolution and human rights tutelage (Hincapié Jiménez and López Pacheco 2014). Amongst indigenous populations, *yaku mama* (mother water) is coupled with the *pachamama* concept to further edify the importance of water into their respective constitutions (Storini and Quizhpe-Gualán 2020). The final aim of these pieces of law is to foster equity, social justice, and pluralism by means of ecological preservation (Iacovino 2020). However, a key step for future legislative amendments is to ensure real political participation (Bejarano and Segura 2013).

Different Latin American constitutions explicitly mention water governance in dedicated articles—see the Guatemalan (Constitution of the Republic Guatemala 1993) and Venezuelan (Constitution of the Republic of Venezuela 1999) constitutions. The Ecuadorian constitution establishes that water is amongst the main ecosystems sources to be protected (art. 127, Constitution of the Republic of Ecuador 2008) but also in this respect, the Ecuadorian and the Bolivian constitutions benchmark. Article 318 even affirms that water is inalienable state property. The Ecuadorian constitution states that energy sovereignty shall not interfere with food sovereignty and the right to water (art. 14). In defining the well-living rights, the first section is devoted to water and food (Title II, Chapter II). Additionally, the Ecuadorian constitution guarantees access to water and quality of water (art. 276; Wolkmer 2018a, b). The Bolivian constitution envisages water amongst the diverse resources pertaining to the natural heritage (Constitution of the Republic of Bolivia 2008, art. 136). The Bolivian constitution also puts into force the right to water as part of the Andean cosmovision (art. 7; Wolkmer 2018a, b). Article 373 is implied as a reaction to the water happened in Cochabamba, Bolivia, in 2000. It prohibits the private appropriation of water, which “constitutes a very fundamental right for life, in the frame of people sovereignty” (see Wolkmer et al. 2019).

#### Water governance in Italy

Italy is one of the countries where water utilities management options have been largely debated in both the political and scholarly environments (Carrozza and Fantini 2013). As a result, legislation has often seen the juxtaposition of public and private management (Landriani et al. 2019). However, multidimensional performance assessments based on sustainability criteria shall be the keystone for progress in water management (Agovino et al. 2021). In this context, a lead role was attributed to stakeholders in line with a multi-stakeholder approach (Lafaye and Crétois 2015; Gatto 2020).

In Naples, a notable action in this direction has been the switch from the corporate ARIN SpA (Azienda Risorse Idriche Napoli) to the public firm ABC Napoli (Acqua Bene Comune, Water as a Commons). The re-municipalization of the Neapolitan water utility stems from legal procedures, scientific production, and local civic movements. This happened following Naples municipal council deliberation n. 740—which took place on June 16, 2011—only three days after the national popular referendum (Louvin 2016; Mone 2016). ABC company statute foresees an “ecological and participative governance,” a “tiny parliament where users, workers, environmentalists, and the municipality councilors are all represented,” configured as the “first advanced organization of commons governance” (Lucarelli and Morand-Deville 2014; Lafaye and Crétois 2015). In Italy, these actions culminated in the 2011 national popular referendum on water. The referendum established limits on water privatization and enlarged space for public and third-sector activities for promoting water as a human right and a commons.

## Discussion: The pursuit of sustainability—towards polycentric and resilient governance for social-ecological systems

### Governing the commons—from theory to practice

In recent years, the study of the options related to the governance of the commons followed economic, legal, and policy points of view. The outstanding caveats formulated by Garret Hardin in 1968 were last tackled in 1990, when Elinor Ostrom conceptualized a new theory to govern the commons, leading to proposals to overcome the issue. Nevertheless, the economic and multidisciplinary analyses carried on by Elinor and Vincent Ostrom and further scholars have come a long way in addressing the issues raised by the commons governance (Frischmann et al. 2019). This theory conceived the adoption of a range of rules concerning the effective regulation and the optimal management of such groups, which would have led to the fruitful use and share of commons. Elinor Ostrom’s theory was supported by empirical evidence of successful and failing communities, that applied such types of rules in real dynamics coming from real societies in addition to a formalization following a strategic approach (Ostrom 1990; Schalager and Ostrom 1999).

Ostrom’s finding demonstrated that the environment and resources are not destined for depletion. Instead, there are multiple solutions to governance, in a polycentric resilient view targeting sustainable commons and natural resource use. These models would involve the public and private sector together with civil society. The solutions furnished by Ostrom’s theory implied the strict adoption to which the users of the commons were called to respect. Good governance and cooperation would have allowed for the maximization of social welfare and long-term equity—of crucial importance for water governance (Zwarteveen et al. 2017).

The arguments presented throughout the current paper at hand aimed to highlight the transition from the pure theoretical evolution into the legislative and practical application of the commons governance with the goal of recognizing distinguished elements for future regulation, adoption, and formal institutionalization offered by diverse methods and approaches. This work emphasized the role of water in both the Latin American and Italian context. In the former, water has been clearly previewed as part of a wider commons defense.

In both cases, the right to water has been gaining a renewed consensus, although more stringent actions to protect water and further commons still need to be undertaken (Zanotelli 2010; Carrozza and Fantini 2016; Wolkmer and Melo, 2012, 2020). This is the case for local indigenous communities and vulnerable socioeconomic groups, who would benefit from engaging with standards and parameters such as ecosystem services for decision-making (Gómez-Betancur and Torres 2021). In the Italian case, more practical pieces of evidence emerged—above all, the Italian public referendum against the privatization of water governance in 2011, the implementation of commons department within Naples municipality, and the municipalization of new ecological and participatory conversion of the Neapolitan local water utility. The water tutelage pathway in Italy has been convoluted and passed by the popular referendum and eventual defense of the outcoming results and the transition towards more participative models of the commons.

The case of water governance in Naples may be considered as an avant-garde of both sustainable development implementation and commons rethinking (Hannachi et al. 2017; Landriani et al. 2019). It is true that the de-corporatization of the water public utility has brought social and environmental results more than economic outputs. While the transition towards participative models has been both an expression of popular willingness—after the 2001 popular referendum—and a propulsor for rejuvenating and stimulating the commons debate, furnishing novel shapes to the issue, hence “re-creating a (new) commons” (Hannachi et al. 2017).

Regarding law perspectives, an empirical laboratory—or even a benchmark—can be detected in the European Union: there, a proliferation of regulations and various legal interventions in resource-related issues, involving multiple actors, have emerged at different levels (Bulkeley and Kern 2006; Ostrom 2010b; Gatto and Busato 2020).

Examining regional applications the new EU legislation should pass a revisited competence allocation with regulatory functions while transitioning from competing jurisdictions to competing competencies (van Zeben 2014). This step will be in line with a polycentric, multilevel governance approach that will eventually enforce subsidiarity and self-governance for governing large multi-jurisdictional, multi-issue systems—intended as sub-dimensions of complex systems. This processwill have to be juxtaposed to an approach based on the EU governance centralization and its outputs (van Zeben 2013).

### The role of resilience policy in facing systems complexity and addressing polycentric governance

A vast literature explores the interconnectedness between adaptive capacity, mitigation, and resilience (Folke 2007; Holling and Gunderson 2002; Holling 2001). The interpretations also refer to addressing biosphere-based sustainability, being grounded on ecological precepts (Folke et al. 2016). The model is strictly intertwined with resilience due to the capacity of social-ecological systems to “absorb both natural and human disturbance while still maintaining structure and function” (Chaffin et al. 2014).

Dynamic formulations of polycentric governance are at the base of effective and sustainable commons provisions, in a framework characterized by a multitude of approaches to address systems complexity (Ostrom 2010a). Polycentricity implies diversified layers of decentralized, connected, and redundant-in-function governance (Andersson and Ostrom 2008; Chaffin et al. 2014). The conceptualization includes overlapping jurisdictions and calls for adaptive governance (McGinnis 1999; da Silveira and Richards 2013).

For polycentricity to function within adaptive governance, institutions—at all levels—are supposed to be nested in different locations and diverse (Dietz et al. 2003; McGinnis and Walker, 2010). For the diverse governance levels and criteria to work, it is required to lead a polycentric approach, embedding simultaneous change of institutions, stakeholders, norms, and (resource) infrastructure (Goldthau 2014).

Polycentric governance has practical, empirical, and legal implications. A relevant practical case is the Amazon governance case—that consolidated multilevel local governance (Chaffin et al. 2014). While in Europe, an important piece of law can be found in the Directive 2009/29/EC—enforced by the European Parliament and the Council to “improve and extend the European Emissions Trading Scheme”, which proposes a decentralization of regulatory competencies in law-making related to climate change (Andersson and Ostrom 2008; van Zeben 2014, 2019).

### Economic, legal, and political implications

The portrayed political economy and ecology pattern requires a triangulation between the economic, legal, and political spheres. Local-to-global scrutiny can be used as a valuable lens where community governance experiences in diverse fields became instructive for modeling the general conceptual formulation (Ostrom et al. 1999; Ostrom and McKean 2001)—an approach notably taken over by Ostrom (1990). There, local communities take a lead role while intervening in fostering solutions,agreements, and cooperation for common sustainability goals—paramount for the emergence of sound regional environmental and natural resource governance strategies, as well as for effective policies (Conca 2012; Balsiger and Prys 2016).

Notable law implications arise where economic cooperation and legal protection call for governance actions. In this sense, collective action issues have the chance to be solved by polycentric systems—grounded in adaptive governance and resilience—within the context of a change in social-environmental systems (Ostrom 2008, 2010b). Governing the commons on sustainable development grounds implies consideration of the polycentric governance for building resilient communities—characterized by an adaptive capacity to change (Gupta et al. 2010).

The illustrated scenario foresees osmosis amongst the economic, legal, and political dimensions while implying novel political economy and ecology pathways (Marella 2017). In this conception, the call for resilient governance in complex systems arises. Resilient governance facilitates tackling vulnerability in natural resources systems, their adaptation and the regulation of transition processes (Gatto and Busato 2020).

## Conclusion, policy solutions, and prospects

Elinor Ostrom asserted the unsuccessfulness of the monistic governance from the market or the state in addressing a sustainable use of natural resource systems (Ostrom, 1990, 2010a). This conclusion implies a strong role for the commons in detecting strategies and solutions to achieve sustainable development targets. In this framework, the public sector, the private sector, and civil society are able to interact. The result of their interplays are improved mutual benefits for all—encompassed in defined and tailored models, which construct a multitude of governance solutions with pluralistic interpretations (Ostrom 2010a; Gatto 2020).

The last legal progress and game theory applications provide fresh policy tools and pose new challenges to the governance of commons. These novelties might embody a relevant role for upcoming research in development, resource economics, and political ecology, using the economic analysis of law, legal research, and social-ecological systems analysis. Furthermore, this may benefit development practices and lawmaking while allowing policymakers to better address the sustainable development agenda and the global policy mandate (UN 2015; Melo and Gatto 2014).

It is true that most of the policy implications coming from the recent agenda priorities structured for international development do not explicitly embed the governance of the commons, SDGs’ goals and targets (UN 2015). Nevertheless, SDGs attribute a primary role to the local commons (especially water, food and agriculture, health, education, and energy) and global commons (thus, in a wider sense, also oceans, atmosphere, space, cyberspace, and climate). These are often referred to as global public goods (Deneulin and Townsend 2007; Karlsson-Vinkhuyzen 2012). In particular, the current climate change prospects require urgent policy questions to be addressed where polycentric, resilient approaches for the commons governance pose multiple doubts on the old-fashion tragedy of the commons (Paavola 2011; Patt 2017). Following the prescribed remedies, SDGs’ design policies tailored actions to govern the commons where a blueprint of actions would be necessary steps to make them work (UN 2015). This process is meant to be threefold, based on social, ecological, and relational inclusiveness (Gupta and Vegelin 2016).

The conceptual, theoretical, and policy underpinnings of the current work may be considered as a lens for elucidating and analyzing emerging ecological economics using sustainable development models and regulations. This is the case for related cooperative games focusing on circular economy (see Bimonte et al. 2021) and metrics gauging environmental innovation and international environmental agreements (Barra et al. 2019). This exercise may also be used as a baseline for prospective law and economics studies, combining game theory with regulatory investigations—which proved effective tools to examine those themes. Additionally, the inquiry at hand may hopefully contribute to inspiring the formulation of new policy and regulation models in addition to informing future civic action. The outlined substructure may be interpreted through different viewpoints, interpretations, theories, techniques, and practices leading to diverse policy implications.

This paper’s findings sort from the intersection of concepts, theories, methodologies, and case studies on the commons. The interlinkages between resilient, polycentric, and reflexive governance is original and is corroborated by the cases of water governance and practice in Latin American Constitutions and Italian utilities management and legal doctrine. This may pave the way for new pluralistic inquiries entangling the mentioned holistic conceptual, theoretical, methodological or policy views on the commons governance.

## Footnotes

1 *Beni demaniali* and *beni patrimoniali.*
